# The phosphatase Shp1 interacts with and dephosphorylates cortactin to inhibit invadopodia function

**DOI:** 10.1186/s12964-021-00747-6

**Published:** 2021-06-04

**Authors:** Alessia Varone, Chiara Amoruso, Marcello Monti, Manpreet Patheja, Adelaide Greco, Luigi Auletta, Antonella Zannetti, Daniela Corda

**Affiliations:** 1 Institute of Biochemistry and Cell Biology, National Research Council, Via Pietro Castellino 111, 80131 Naples, Italy; 2 Interdipartimental Center of Veterinary Radiology, University of Naples Federico II, Via Delpino 1, 80137 Naples, Italy; 3 Institute of Biostructures and Bioimaging, National Research Council, Via Tommaso De Amicis 95, 80145 Naples, Italy; 4 IRCCS SDN, Via Emanuele Gianturco 113, 80142 Naples, Italy; 5 Department of Biomedical Sciences, National Research Council, Piazzale Aldo Moro 7, 00185 Rome, Italy

**Keywords:** Src homology region 2 domain-containing phosphatase-1 (Shp1), Cortactin, Invadopodia, Glycerophosphoinositols, Phosphoinositides, Cancer

## Abstract

**Background:**

Invadopodia are actin-based cell-membrane protrusions associated with the extracellular matrix degradation accompanying cancer invasion. The elucidation of the molecular mechanisms leading to invadopodia formation and activity is central for the prevention of tumor spreading and growth. Protein tyrosine kinases such as Src are known to regulate invadopodia assembly, little is however known on the role of protein tyrosine phosphatases in this process. Among these enzymes, we have selected the tyrosine phosphatase Shp1 to investigate its potential role in invadopodia assembly, due to its involvement in cancer development.

**Methods:**

Co-immunoprecipitation and immunofluorescence studies were employed to identify novel substrate/s of Shp1AQ controlling invadopodia activity. The phosphorylation level of cortactin, the Shp1 substrate identified in this study, was assessed by immunoprecipitation, in vitro phosphatase and western blot assays. Short interference RNA and a catalytically-dead mutant of Shp1 expressed in A375MM melanoma cells were used to evaluate the role of the specific Shp1-mediated dephosphorylation of cortactin. The anti-invasive proprieties of glycerophosphoinositol, that directly binds and regulates Shp1, were investigated by extracellular matrix degradation assays and in vivo mouse model of metastasis.

**Results:**

The data show that Shp1 was recruited to invadopodia and promoted the dephosphorylation of cortactin at tyrosine 421, leading to an attenuated capacity of melanoma cancer cells to degrade the extracellular matrix. Controls included the use of short interference RNA and catalytically-dead mutant that prevented the dephosphorylation of cortactin and hence the decrease the extracellular matrix degradation by melanoma cells. In addition, the phosphoinositide metabolite glycerophosphoinositol facilitated the localization of Shp1 at invadopodia hence promoting cortactin dephosphorylation. This impaired invadopodia function and tumor dissemination both in vitro and in an in vivo model of melanomas.

**Conclusion:**

The main finding here reported is that cortactin is a specific substrate of the tyrosine phosphatase Shp1 and that its phosphorylation/dephosphorylation affects invadopodia formation and, as a consequence, the ability of melanoma cells to invade the extracellular matrix. Shp1 can thus be considered as a regulator of melanoma cell invasiveness and a potential target for antimetastatic drugs.

**Video abstract**

**Supplementary Information:**

The online version contains supplementary material available at 10.1186/s12964-021-00747-6.

## Background

Tumor progression is dependent on the intrinsic properties of cancer cells, such as their ability to detach from the primary site, invade the surrounding tissues and colonize secondary sites, thus spreading tumors to distal organs and form metastases [1]. The initial steps of the metastatic process include the local invasion of cancerous cells into the extracellular matrix (ECM) and connective tissue with the subsequent penetration into the vascular and lymphatic systems. This invasive process is driven by the reorganization of the actin cytoskeleton and formation of cell structures, the invadopodia [2].

These are actin-rich protrusions of the plasma membrane, formed at the adherent surface of invading tumor cells. A range of components are recruited to these structures, including proteins involved in the enzymatic degradation of the ECM, as well as in the actin and membrane remodeling. Invadopodia develop through a series of maturation events, which include the formation of a central-filamentous actin core, the invadopodium precursor, followed by actin polymerization that pushes the plasma membrane outward, and the recruitment of secreted and membrane-bound matrix metalloproteases (MMPs) [3].

The temporal sequence of events that drive invadopodia formation and function is initiated in response to signals triggered by growth factors or matrix receptors, oncogenic transformation, heterotypic cell interaction, epithelial–mesenchymal transition (EMT) or MMP activity [4]. While the structural components of invadopodia have been well studied and characterized, the comprehension of the regulation of invadopodia dynamics is still incomplete.

Tyrosine phosphorylation is a major regulator of invadopodia formation and function, affecting all stages of invadopodia life and allowing the spatiotemporal control of their activity. In this context, several protein tyrosine kinases (PTKs) are important for initiation of invadopodia assembly and function, as it is the case of Src and Arg kinases [5]. In contrast, the role of protein tyrosine phosphatases (PTPs) in the formation of invadopodia remains unclear.

We have addressed this issue by testing the involvement of the Src-homology region 2 (SH2) domain-containing phosphatase 1 (Shp1) in invadopodia formation and ECM degradation, as it has a well-known role in the regulation of Src kinase [6].

Shp1 is a non-receptor protein tyrosine phosphatase that acts as a negative regulator of inflammation [7]; it is mainly expressed in hematopoietic and epithelial cells and is widely accepted as a negative regulator of signaling pathways involving cell proliferation, differentiation, survival, apoptosis and adhesion [8]. However, the precise function and targets of Shp1 in non-hematopoietic cells are largely unknown [9]. Numerous studies have proposed Shp1 as a candidate tumor suppressor gene in lymphoma, leukaemia and several solid cancers, as it functions as an antagonist of the growth-promoting and oncogenic potentials of tyrosine kinases [10, 11]. The involvement of Shp1 in cancer progression has been also supported by the notion that this phosphatase results to be down-regulated or absent in various cancer cell lines and tissues [12–14].

Shp1 contains two SH2 domains at the N-terminal, a catalytic phosphatase domain and a C-terminal tail [15]. The biochemical and structural characterization of Shp1 clearly reveals an auto-regulation via an inhibitory rearrangement of the protein, in which the insertion of the N-terminal SH2 domain into the phosphatase domain blocks the enzymatic activity of Shp1. The binding of the SH2 domains to specific tyrosine phosphorylation sites on different molecules leads to structural rearrangements of Shp1, with the consequent release of the catalytic site and fully activation of the protein [15].

We have previously demonstrated that the phosphatase Shp1 is an intracellular receptor of the phosphoinositide-derived cell mediator glycerophosphoinositol 4-phosphate (GroPIns4*P*) [16]. In NIH3T3 fibroblasts GroPIns4*P* endogenously-formed by EGF receptor-activated cPLA_2_α, binds to the Shp1-SH2 domain, starting the Src-dependent signaling cascade that promotes ruffle formation and stimulation of cell motility [16–19]. Interestingly, GroPIns4*P* along with its unphosphorylated form (glycerophosphoinositol (GroPIns)) when added exogenously, shows pharmacological effects relevant to tumor spreading: both compounds inhibit migration through the ECM of human melanoma (A375MM) cells and human mammary carcinoma (MDA-MB-231) cells most likely affecting invadopodia function [20].

In this study, we investigate the involvement of Shp1 in cancer invasion, report its localization at invadopodia, and define how Shp1 reduces ECM degradation through a direct, yet unknown, effect on cortactin phosphorylation at invadopodia. We also explore the GroPIns-dependent regulation of invadopodia function mediated by its binding to Shp1.

## Methods

### Antibodies, constructs and reagents

The antibodies used were: anti-cortactin (p80/85), clone 4F11 (Merck-Millipore, Darmstadt, Germany); anti-pTyr421 and anti-pTyr466-cortactin (Sigma-Aldrich, WI, USA); anti-Shp1, anti-Nck1 and anti pSer505-cPLA_2_ (Santa Cruz, CA, USA); fluorophore-conjugated antibodies and fluorophore-conjugated actin (Molecular Probes, Oregon, USA); anti-actin and anti-HRP (Abcam, Cambridge, UK).

The expression vectors used were: Shp1 and Shp1-C455S (F.D. Böhmer, CMB, Friedrich Schiller University of Jena, Germany). The glycerophosphoinositols were either obtained from Echelon Biosciences Inc. (UT, USA) or kindly provided by Euticals S.p.A (Italy).

### Cell culture, cDNA and siRNA transfection

The human melanoma A375MM were grown under standard conditions, as previously described [20]. Cells were plated at 50% confluence in 6-well plates and transfected using Lipofectamine LTX (Invitrogen, CA, USA), according to the manufacturer’s instructions. For RNA interference, cells were transfected with 200 nM of the siGENOME™ SMARTpool® reagents (Dharmacon, Lafayette, CA, USA) containing four pooled siRNA duplexes against human Shp1 using Lipofectamine RNAiMAX (Invitrogen, CA, USA) according to the manufacturer’s instructions. The cells were plated on gelatin-coated coverslips 48 h after siRNA treatment in the presence of 5 μM BB94, a broad-range matrix metalloprotease inhibitor (British Biotech, UK) and incubated at 37 °C in the presence of 5% CO2 for an additional 24 h. The ECM degradation and invadopodia formation were evaluated as described below.

### Immunofluorescence

A375MM cells were grown on gelatin-coated coverslips, fixed with 4% paraformaldehyde for 10 min at RT and then washed three times in PBS. The blocking reagent (0.05% saponin, 0.5% BSA, 50 mM NH_4_Cl) was added to cells for 20 min, followed by 1 h or O/N incubation with the primary antibodies in the blocking reagent. Cells were then washed with PBS and incubated with the secondary antibodies (1:400) for 45 min. The coverslips were then mounted on glass microscope slides with Mowiol. For the staining of Shp1 at the invadopodia, cells were fixed with ice-cold methanol for 5 min at − 20 °C, washed and then permeabilized in PBS containing 0.5% Triton for 10 min. Cells were subsequently processed for immunofluorescence microscopy. Confocal images were acquired using a Zeiss LSM700 inverted confocal microscope system (Carl Zeiss, Gottingen, Germany). Fixed cells were analyzed using a 63 × oil-immersion objective, maintaining the pinhole of the objective at 1 Airy unit. Fluorescence intensity was calculated by integration of the IF signal within the region of interest divided by the area. The intensity of cortactin and Y421-cortactin phosphorylation staining was quantified and expressed as the relative fold change in pY421-cortactin/cortactin ratio.

### ECM degradation assay

Fluorescent gelatin-coated coverslips were prepared and the ECM degradation assay was performed according to the previously published protocol with some modifications [20]. Briefly, cells were plated on gelatin-coated coverslips, in medium containing 5 μM BB94. After 16 h, BB94 was washed out to allow synchronous invadopodia formation, and cells were fixed after 3 h and processed for immunofluorescence. For transfection cells were plated at 50% confluence in six-well plates and transfected the following day, as described above. Six h after transfection, cells were detached, plated on gelatin-coated coverslips for 16 h and processed as described above. Degradation area was quantified analyzing the area of ECM devoid of fluorescence as described previously [21].

### Proximity ligation assay

Proximity ligation assays were performed using the Duolink anti-Mouse MINUS and anti-Rabbit PLUS in situ PLA probes and the Duolink in situ Detection Reagents Red (Olink Bioscience), following the manufacturer's instructions. The amplified signals were analyzed using a Zeiss LSM700 inverted confocal microscope system (Carl Zeiss, Gottingen, Germany).

### Western blotting, immunoprecipitation and pull-down

A375MM cells were washed with ice-cold PBS and lysed on ice in buffer containing 20 mM Tris–HCl, pH 8.0, 150 mM NaCl, 1% Triton-X100, 5 mM Na_3_VO_4_, 30 mM β-glycerophosphate and 10 mM NaF supplemented with the protease inhibitor cocktail (Complete Mini EDTA-free, Roche) and analyzed by SDS-PAGE. Western blotting was performed with the indicated antibodies. For Shp1 immunoprecipitation, 1 mg lysate protein from A375MM cells was incubated with 2 μg anti-Shp1 polyclonal antibody (O/N, 4 °C, shaking). Then 50 μl protein A Sepharose beads were added for a further 1 h incubation (4 °C, shaking). For cortactin immunoprecipitation, 40 μl anti-FLAG M2 agarose beads were added to 1 mg of A375MM cell lysates (O/N, 4 °C, shaking). Western blotting was performed from the washed and denatured complexes. For His-Shp1 pull-down assay, cortactin was immunoprecipitated from A375MM cell lysates as described above. The immunoprecipitates were washed three times with lysis buffer and 3 μg His-Shp1 was added to each sample in absence or presence of 50 μM of GroPIns or GroPIns4*P* (1 h, 4 °C, shaking). The beads were then washed three times with lysis and the bound protein was eluted from anti-FLAG M2 agarose beads by boiling (10 min) in 100 μl SDS-sample buffer. For GroPIns-Bio pull-down assay with purified Shp1, 1 μg of purified Shp1 was incubated for 16 h at 4 °C with 1 mg of streptavidin- conjugated paramagnetic beads in the presence of 2.5 nmoles of biotin or GroPIns-Bio in binding buffer (50 mM Tris–HCl, pH 7.6, 50 mM KCl, 10 mM EDTA) plus proteases inhibitors. Following this incubation, the unbound material was removed and beads were washed with binding buffer. The beads with bound protein were boiled in 100 μl of SDS-sample buffer.

### Invadopodia fractionation

Purification of an invadopodia-enriched sub-cellular fraction was performed as published [22]. A375MM cells were cultured at 3.5 million cells/25 cm Petri dishes on cross-linked gelatin. After 24 h, when cells were about 70/80% confluent, plates were first washed in PBS containing 0.5 mM MgCl_2_, 1 mM CaCl_2_, then in five-times diluted PBS containing 0.5 mM MgCl_2_, 1 mM CaCl_2_, and incubated for 15 min with 3 ml of the diluted PBS containing a protease inhibitor mixture to induce cell swelling. Cell bodies were then sheared away using an L-shaped Pasteur pipette with sealed end, to leave invadopodia remnants embedded in the gelatin. These were then washed in PBS containing 0.5 mM MgCl_2_, 1 mM CaCl_2_ before being scraped away with the cross-linked gelatin into RIPA buffer (150 mM NaCl, 1% NP40, 0.5% sodium deoxycholate, 0.1% sodium dodecyl sulphate, 50 mM Tris base buffer pH 8.0, proteases inhibitors) and clarified by centrifugation (15 min, 13,000 rpm at 4 °C). The cell body fraction was further separated into the cell body membranes and cytosol fractions by centrifugation at 9000 × g for 20 min at 4 °C. The supernatant (cytosolic fraction) was used directly whereas the cell-body membrane pellet obtained after centrifugation was solubilized in RIPA buffer and clarified by centrifugation (15 min, 13,000 rpm at 4 °C). The whole lysate was obtained from cells grown in 10 cm Petri dishes on cross-linked gelatin. Cells were plated at 2 × 10^6^ cells/10 cm dish, washed after 24 h with PBS buffer containing 0.5 mM MgCl_2_, 1 mM CaCl_2_, and finally solubilized in RIPA buffer and clarified by centrifugation (15 min, 13,000 rpm at 4 °C).

### In vitro dephosphorylation of cortactin

Cortactin was immunoprecipitated from A375MM cells as described above. The immunoprecipitates were washed three times with lysis buffer, three times with lysis buffer without phosphatases inhibitors, and twice with phosphatase buffer (100 mM Na-Hepes, pH 7.4, 150 mM NaCl, 1 mM EDTA, and 10 mM DTT). The immunoprecipitates were then split equally into different tubes for treatment with Shp1 or buffer only. The treatment consisted of the addition of 2 µg Shp1 to the immunoprecipitates followed by incubation at 37 °C for different times. The reactions were terminated by the addition of concentrated SDS-sample buffer. The samples were then analyzed by SDS-PAGE, and the level of Tyr421 and Tyr470 phosphorylation was determined by western blotting and normalized for the total amount of immunoprecipitated cortactin.

### Expression and purification of recombinant Shp1

Recombinant purified His-Shp1 was produced from *E. Coli* BL21(DE3) transformed cells with pETM11-Shp1, grown at A_600_ = 0.6 before induction with 0.1 mM IPTG for 16 h at 20 °C. Cells were then harvested by centrifugation at 6,000 rpm for 10 min and rinsed twice with PBS. The pellet was re-suspended in lysis buffer (25 mM Tris–HCl, pH 7.5, 150 mM NaCl, 10 mM β-mercaptoethanol, 20 mM imidazole) containing protease inhibitor cocktail as described above, and lysozyme. The suspension was incubated at 4 °C for 30 min and sonicated on ice 8 times for 15 s. Subsequently Triton X-100 was added to a final concentration of 1% w/v, and the mixture was incubated for 15 min at 4 °C. The bacterial lysate was then centrifuged at 22,000 rpm for 30 min at 4 °C. The supernatant was added to a Ni–NTA–agarose column that had been previously equilibrated in lysis buffer for 3 h at 4 °C. After elution with lysis buffer containing 250 mM imidazole, His-Shp1 was dialyzed twice against 1000 volumes of PBS and stored in aliquots at − 80 °C.

### Animal models and treatments

All in vivo experimental procedures were performed after approval of the Italian Ministry of Health (Protocol N 38/2015) and in accordance with the European guidelines of the 2010/63/EU Directive and with National Institutes of Health (NIH) recommendations, at the CEINGE, Biotecnologie Avanzate s.c.a.r.l. All efforts were made to minimize animal suffering and the number of animals necessary to produce reliable results. The animal procedures were performed under inhalant general anesthesia (isoflurane in oxygen at 0.8 Lt/min). The lung metastatic model was developed as previously described [23]. Briefly, 5-week old BALB/c nude mice were injected intravenously with 2 × 10^6^ of wild-type or Shp1-kd A375MM cells in 100 µl medium, and immediately treated with either GroPIns (50 mg/kg) or vehicle (PBS) intraperitoneally once per day, 5 days per week for two consecutive weeks.

### Fluorescence reflectance imaging

All mice were maintained on a diet with a purified, alfalfa-free rodent chow for 15 days before fluorescence imaging to minimize fluorescence in the gut. The metastatic model was analyzed by Fluorescent Reflectance Imaging (FRI) at the end of the 2-weeks therapy period as previously reported [24]. Each mouse, under isoflurane anesthesia, was injected intravenously with 2 nmol of ProSense 750 (a near infrared imaging cathepsin-activatable agent) resuspended in 100 µl PBS, through a PE10 tubing connected to a 30G needle. Mice were analyzed at different time points by FMT 4000 imaging system (PerkinElmer, Waltham, MA). Two-dimensional acquisitions were performed at 2 h and 24 h post-injection. After the last acquisition, animals were euthanized while still under anesthesia and lungs were removed and imaged ex vivo. All acquisitions were studied off-line, drawing a region of interest (ROI) to include the lungs, and semi-quantitative (counts/energy) data were collected for each mouse. Epifluorescence (2D) datasets were both acquired and analyzed by FMT system software (TrueQuantTM v4.0) from PerkinElmer (Waltham, MA).

### Generation of A375MM Shp1-kd cell line

The A375MM Shp1-kd cell line was generated by the CRISPr/Cas9 system. The sgRNA sequence (5′-GAGTACTACACTCAGCAGCA-3′) has been selected using the GPP (Genetic Perturbation Platform) sgRNA Designer tool of the Broad Institute as the one with higher score between sgRNAs within the first exons of the *ptpn6* gene. The sgRNA was cloned into the pSpCas9(BB)-2A-GFP (PX458) vector (plasmid #48138, Addgene) by using the *BbsI* restriction enzyme to digest the vector and the T4 DNA ligase to incorporate the sgRNA into the vector. Minipreps were performed on colonies grown on ampicillin plates and the insertion of the sgRNA into the plasmid was confirmed by sequencing with U6 promoter primer (5′-GAGGGCCTATTTCCCATGATTCC-3′). A375MM cells were transfected with 2.5 µg of pSpCas9(BB)-2A-GFP/*ptpn6* sgRNA using Lipofectamine LTX (Invitrogen, CA, USA). After 48 h, cells were sorted for GFP expression with BD FACSAria III flow cytometer selecting one cell in each well of 96-well plate to generate a specific clone. Cells were amplified and Shp1 transcriptional levels, as well as protein expression, were evaluated by qRT-PCR and western blot analyses, respectively.

### qRT-PCR

RNA was extracted by using the RNeasy Mini Kit (Cat. No. 74106, Qiagen) and 1 µg was reverse transcripted following the instructions of the QuantiTect Reverse Transcription Kit (Cat. No. 205313, Qiagen). One hundred ng of cDNA were amplified by using the Syber Green Master Mix (Applied Biosystems) and the specific primers for *ptpn6* (forward 5′-AGAGATGCTGTCCCGTGGGT-3′ and reverse 5′-AAGTCACCCTGGTTCTTGCG-3′). Light Cycler 480 II thermocycler (Roche) was used for the reaction and CT data analysis. The relative gene expression was calculated by using 2^ (− delta delta CT) method and GAPDH as housekeeping gene.

## Results

### Shp1 is localized at invadopodia in melanoma A375MM cells

Invadopodia are composed of multiple cytoskeletal, trafficking and signaling proteins and are recognized by the colocalization of actin, cortactin, phosphotyrosine among other proteins, with areas of matrix degradation [25]. Based on previous studies showing a role of Shp1 in cancer progression and in EMT [26–29], as well as its association with cytoskeleton components [16, 30, 31], we hypothesized a Shp1 involvement in invadopodia formation/regulation.

**Fig. 1:**
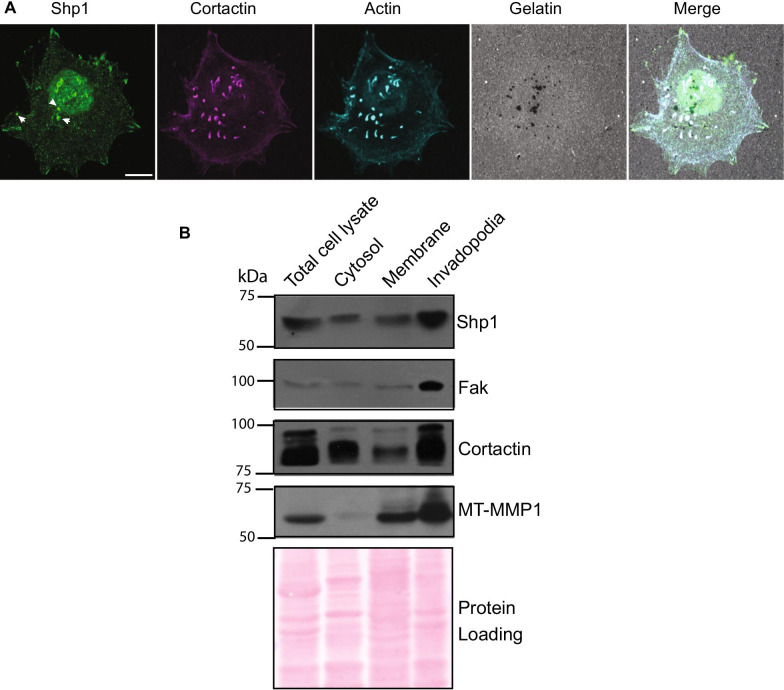
Shp1 localizes at invadopodia in A375MM cells. **A** Representative confocal images of A375MM cells showing colocalization of Shp1 with invadopodium markers. Cells were plated on fluorescent gelatin-coated coverslips in complete media, fixed and immunostained for cortactin and actin. Shp1, cortactin, actin and gelatin are shown in green, magenta, cyan and gray, respectively. Degraded ECM is shown as dark areas. The invadopodia are shown as cortactin- and actin-positive dots corresponding to the dark areas. Arrowheads indicate invadopodia colocalizing with Shp1. Confocal images are representative of at least four different experiments. Scale bars, 10 μm. **B** Shp1, Fak, cortactin and MT1-MMP protein expression were assayed by western blotting in cytosolic, plasma membrane and invadopodia-enriched subcellular fractions extracted from lysates of A375MM cells incubated for 24 h on a thick layer of cross-linked gelatin, as described in “Methods” section. The invadopodia markers cortactin and MT1-MMP were enriched in the invadopodia fraction. Protein loading was assessed by Ponceau red staining. Molecular weight standards (kDa) are indicated on the left of each panel. Representative western blot images of two independent experiments

Thus, we first analyzed the intracellular localization of endogenous Shp1 in human melanoma A375MM cells, a cell model system extensively studied for the invadopodia formation process [32]. In these cells, according to the reported distribution profile, Shp1 is present in the cytosol, plasma membrane, and nucleus [33, 34]. To examine the cytoskeleton-associated fraction we first removed the soluble fraction of Shp1 from A375MM cells permeabilized with Triton X-100 prior to fixation and immunostaining (see “Methods” section); under these conditions, Shp1 was readily observed at invadopodia where it colocalized with cortactin and actin, two well-known invadopodia markers, in approximately 40% of invadopodia-forming cells and it was also detected at focal adhesions located at the cell periphery (Fig. 1A).

To corroborate these findings, we isolated invadopodia-enriched subcellular fractions from A375MM cells plated on cross-linked gelatin-coated dishes, and separated the lysed cells from the invadopodia embedded in the gelatin (see “Methods” section) [22]. To validate the invadopodia enrichment procedure, we tested the different fractions by immuno-blotting for proteins known to localize in invadopodia (cortactin, membrane type 1-matrix metalloproteinase (MT1-MMP)) and adhesion-related protein (focal adhesion kinase, FAK) to ensure that we were isolating the ventral membrane portion of the cell. Shp1 was detected in all fractions analyzed (cell lysates separated into cytosol and membrane, and invadopodia-enriched fraction; Fig. 1B) confirming Shp1 as part of the invadopodia structure, consistently with the localization evidenced above by immunofluorescence (Fig. 1A).

### Shp1 inhibits invadopodia formation and extracellular matrix degradation

Invadopodia assembly and disassembly are central in cancer invasion. These processes are finely regulated by structural and signaling molecules [4]. Having determined the localization of Shp1 at invadopodia, we set out to analyze its possible role in the formation and function of these structures.

**Fig. 2:**
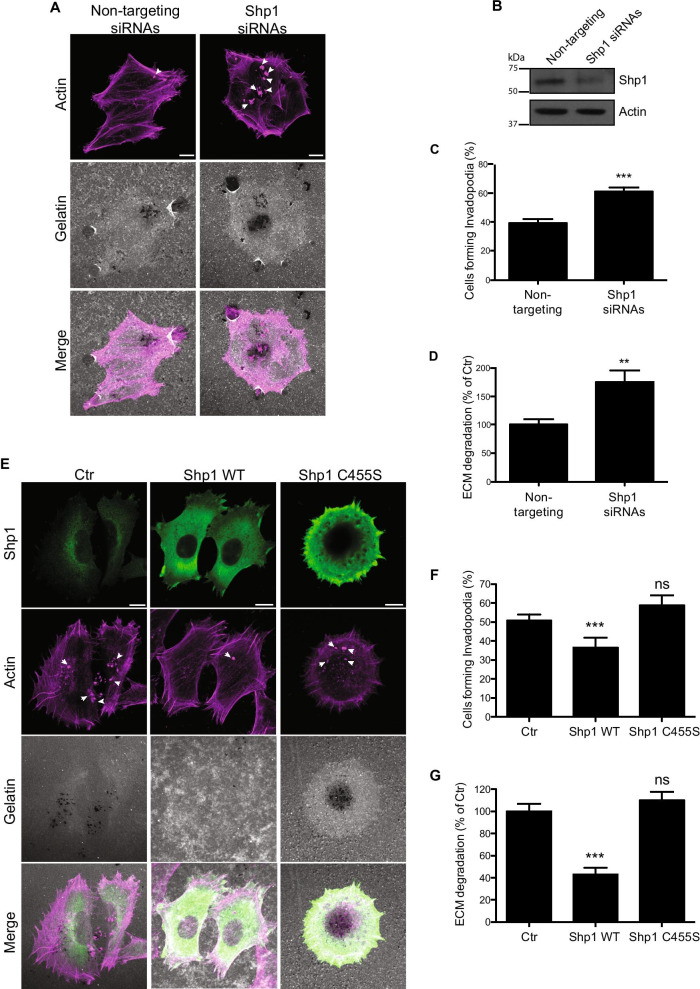
Shp1 regulates invadopodia dynamics in A375MM cells. **A** Representative confocal images of non-targeting siRNAs and Shp1 siRNAs-treated A375MM cells plated on fluorescent gelatin-coated coverslips. Cells were then fixed and stained with phalloidin (magenta). **B** Western blot analysis of Shp1 protein in A375MM cells upon Shp1 knock-down. Actin was used as a loading control. Molecular weight standards (kDa) are indicated on the left the panel. **C**, **D** Quantification of cells forming invadopodia (expressed as percentage of total cells; at least 80 cells were analyzed for each condition) (**C**) and of the total area of degradation (expressed as percentages of control; at least 30 cells were analyzed for each condition) (**D**) of cells treated as in **A**. (**E**) Representative confocal images of A375MM cells transfected with Shp1 WT or the Shp1 C455S mutant and plated on fluorescent gelatin-coated coverslips (gray). Cells were fixed and stained with an anti-Shp1 antibody (green) and phalloidin (magenta). **F**, **G** Quantification of cells forming invadopodia (expressed as percentage of total cells; at least 80 cells were analyzed for each condition) (**F**) and of the total area of ECM degradation (expressed as percentages of control; at least 30 cells were analyzed for each condition) (**G**) of cells treated as in E. Degraded ECM is shown as dark areas on gray gelatin. The invadopodia are shown as actin-positive dots corresponding to the dark areas. Arrowheads indicate active invadopodia. Data are expressed as the means (± SE) of at least three independent experiments performed in duplicate. ***P < 0.001; **P < 0.02; ns *P* > 0.05 (Student’s t-test) calculated for each treatment versus untreated samples. Scale bars, 10 μm

Thus, Shp1 was knocked down in A375MM cells via RNA interference (siRNA; see “Methods” section); cells were then plated on thin rhodamine-labelled gelatin for 16 h to visualize invadopodia-mediated matrix degradation (Fig. 2A). A scramble siRNA was used as a control. Shp1 depletion efficiency was up to 90%, as determined by Western blot analysis (Fig. 2B).

The formation of invadopodia was visualized by staining filamentous actin with fluorescently conjugated phalloidin (see “Methods” section). Silencing of Shp1 resulted in a significant increase in the number of cells forming mature invadopodia (identified by the colocalization of actin dots with ECM degradation area [35, 36]; up to ≈ 50%; Fig. 2C). The function of these invadopodia was then evaluated by quantifying the area of gelatin degradation per cell (visualized as black areas in the gelatin layer): an increase (≈ 70%) in the mean degradation area/cell was detected in Shp1-knockdown cells, indicating a parallel increase in the ability to invade the ECM under these conditions (Fig. 2D). On the opposite, the overexpression of wild-type Shp1 caused a significant suppression of invadopodia formation and, consequently, of ECM degradation (≈ 60% reduction compared to empty vector; Figs. 2E, F) confirming the role of Shp1 in modulating the invadopodia activity.

The overexpression of a Shp1 inactive mutant (in which the mutation of the Cys455 to Ser in the phosphatase domain results in a Shp1 catalytically inactive that also functions as dominant-negative [16, 37–39]; Shp1 C455S) instead gave rise to a phenotype more similar to that of the Shp1 knocked-down cells: the invadopodia formation, as well as the capability to degrade the ECM, were maintained or slightly increased compared to non-transfected cells (Fig. 2E–G). These results define the requirement of the catalytically-active form of Shp1 in regulating invadopodia number and proteolytic activity in A375MM cells.

### Cortactin as a novel Shp1 substrate

Due to the ability of Shp1 to modulate invadopodia formation and function, we investigated the mode of association of this phosphatase with some invadopodia components, *i.e.*, we set at identifying the phosphatase Shp1 substrates in A375MM cells.

**Fig. 3:**
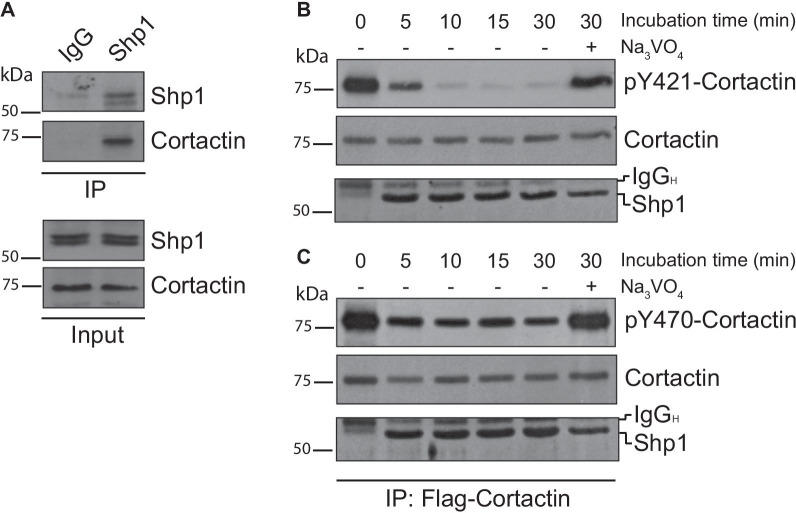
Shp1 interacts with cortactin. **A** Interaction between Shp1 and cortactin was examined by immunoprecipitation with an anti-Shp1 antibody (IP) in A375MM cells plated on gelatin-coated Petri dishes. The expression levels of Shp1 and cortactin were examined in total lysates (input). **B**, **C** Representative immunoprecipitated cortactin fraction (IP: Flag-cortactin) from A375MM cell lysates washed and incubated with purified recombinant Shp1 for the indicated time points at 37 °C in the absence (−) or presence (+) of 5 mM Na_3_VO_4_ (as indicated). The tyrosine-phosphatase inhibitor Na_3_VO_4_ was used as negative control. The top panels show western blot analyses with an anti-pY421-cortactin (**B**) and anti-pY470-cortactin (**C**) antibodies to reveal the specific phosphorylation of tyrosine residues in cortactin. The blots were then re-probed with an anti-cortactin antibody for immunoprecipitated proteins (*middle panels*). The lower panels show purified recombinant Shp1 added in each sample. Molecular weight standards (kDa) are indicated on the left of each panel. IgG_H_, IgG heavy chain; IP, immunoprecipitation

Cells were seeded onto gelatin-coated Petri dishes to evaluate their degradative power, allowed to form invadopodia for 16 h and then lysed and immunoprecipitated with anti-Shp1 polyclonal antibody (see “Methods” section). Immunoprecipitated protein complexes were then analyzed by western blot with antibodies against several known invadopodia components including Src, Fak, Erk1/2, actinin and Cdc42 but only the anti-cortactin antibody recognized the protein that was then found to specifically immunoprecipitate with Shp1 (Fig. 3A).

Cortactin is a key actin regulator and scaffolding protein linking signaling, membrane trafficking and other actin-binding proteins to dynamic actin networks [40]. Cortactin regulates invadopodia functions [35, 41, 42] and its tyrosine phosphorylation represents a critical step in invadopodial maturation, initiating cofilin-dependent barbed-end formation and Arp2/3-dependent actin polymerization [42, 43].

Since tyrosine-phosphorylated proteins interact with Shp1 as putative substrates, we investigated whether Shp1 was able to dephosphorylate cortactin in vitro. Hence, phosphorylated, Flag-tagged cortactin was immunoprecipitated from A375MM cell lysates using anti-Flag beads and then used as a substrate in in vitro phosphatase assays in the presence of purified recombinant Shp1 (see “Methods” section). Cortactin phosphorylation was then assessed using phosphospecific antibodies that recognize phosphorylated tyrosine 421 or 470, the two sites known to regulate invadopodia formation and maturation [44]. Western blot analysis indicated a decrease in the cortactin phosphorylation at tyrosine 421 (pY421-Cortactin) after 5 min of incubation with purified Shp1, which was almost complete after 10 min (Fig. 3B). In contrast, phosphorylation at tyrosine 470 (pY470-Cortactin) was only slightly affected by Shp1, an effect that was however time independent and pointed at the specificity of the Shp1-phosphatase activity, active on the pY421-Cortactin only (Fig. 3C). This conclusion was further supported by experiments using immunopurified tyrosine-phosphorylated Src as a substrate in in vitro phosphatase assays with recombinant Shp1. The phosphorylation of Src at tyrosine 416 (pY416-Src) was monitored by western blotting and, as previously indicated [6], Shp1 was not able to dephosphorylate Src at this specific residue (Additional file 1: Figure S1).

Collectively, these data point at cortactin as a *bona fide* Shp1 substrate whose phosphorylation level is regulated by this phosphatase, possibly affecting invadopodia function.

### Shp1 reduces matrix-degrading invadopodia activity in response to glycerophosphoinositol

Shp1 was previously isolated as a direct-cellular target of the glycerophosphoinositols by pull-down assay coupled with liquid chromatography-tandem mass-spectrometry analysis; it was then shown to mediate the GroPIns4*P*-effects on the actin cytoskeleton in fibroblasts [16, 19]. GroPIns4*P* and its unphosphorylated form GroPIns were also shown to inhibit chemoinvasion of the A375MM melanoma cells, an effect due to a decreased ability of these cells to degrade ECM components [20]. Given this evidence, we hypothesized that Shp1, whose overexpression produces a phenotype similar to that induced by glycerophosphoinositols treatment in A375MM melanoma cells, may be activated by glycerophosphoinositols binding also in cancer cells, thus leading to a reduction in invadopodia function (similarly to the mechanism identified in fibroblasts [16]).

**Fig. 4:**
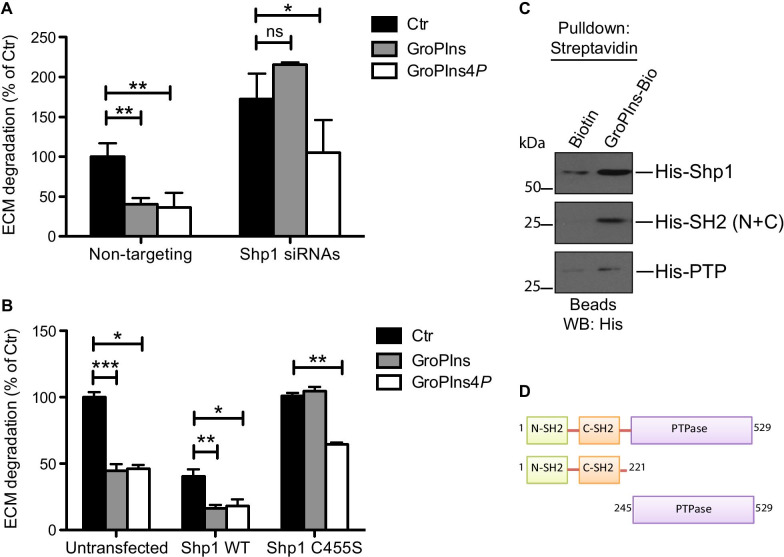
Shp1-induced inhibition of ECM degradation in the presence of the indicated glycerophosphoinositols in A375MM cells. **A** ECM degradation levels evaluated in non-targeting siRNAs and Shp-1 siRNAs-treated A375MM cells plated on gelatin-coated coverslips and incubated in the absence and presence of 50 μM GroPIns and GroPIns4*P* for 16 h. Representative pictures are shown in Additional file 1: Figure S2. **B** ECM degradation levels evaluated in control, Shp1 WT, and Shp1C455S-transfected cells plated on gelatin-coated coverslips and incubated in the absence and presence of 50 μM GroPIns and GroPIns4*P* for 16 h. Representative pictures are shown in Additional file 1: Figure S3. Data are expressed as the means (± SE) of at least three independent experiments performed in duplicate and indicate the total area of degradation (expressed as percentages of control). ***P < 0.001; **P < 0.02; *P < 0.05; ns *P* > 0.05 (Student’s *t*-test) calculated for each treatment *versus* the respective untreated control, as indicated. Note that the *P*-value was also calculated for the other samples to compare the different effects of each treatment according to the presence/absence of Shp1 in the system: * for Non-targeting Ctr *versus* Shp1 siRNAs Ctr; *** for Non-targeting GroPIns *versus* Shp1 siRNAs GroPIns; ns for Non-targeting GroPIns4*P vs* Shp1 siRNAs GroPIns4*P*. **C** Representative pull-down of streptavidin-conjugated beads using Biotin or biotinylated GroPIns (GroPIns-Bio) with either Shp1 1–529 (His-Shp1), the N-terminal SH2-domain mutant (His-SH2 (N + C)) or the catalytic-domain mutant (His-PTPase) of Shp1. Eluted (beads) proteins were analyzed by western blotting using an anti-Histidine antibody. Molecular weights (kDa) are indicated on the left of each panel. **D** Schematic domain structure illustrating the amino acid sequences of the Shp1 mutants used in the pull-down

We first assessed whether Shp1 was involved in the glycerophosphoinositols-mediated inhibition of ECM degradation in A375MM cells. To this end, Shp1-silenced cells were treated with 50 μM GroPIns and GroPIns4*P* respectively, and the invasive potential monitored by measuring the extent of degradation of the fluorophore-conjugated ECM substrate (see above and “Methods” section). In non-targeting siRNAs-treated cells, GroPIns inhibited the total area of ECM degradation by about 60%, while in cells with reduced Shp1 expression levels the GroPIns inhibitory effect was completely abolished (Fig. 4A and Additional file 1: Figure S2). Interestingly, under these conditions the GroPIns4*P* could still inhibit ECM degradation, indicating that it does not modulate the activity of Shp1 leading to ECM degradation, unlike its modulation of the Shp1-Src cascade in fibroblasts (Fig. 4A and [16]).

Next, as a further approach to study the regulation of Shp1 by the glycerophosphoinositols, we analyzed cells overexpressing the active and defective Shp1 mutants. Importantly, while upon overexpression of Shp1 WT the extent of ECM degradation in the presence of GroPIns and GroPIns4*P* was equally inhibited, as compared to non-transfected cells (≈ 60% inhibition in non-transfected and Shp1-overexpressing cells; the latter having a lower degrading activity as already shown above and in Fig. 2), transient expression of catalytically inactive Shp1 C455S mutant completely counteracted the GroPIns modulation of the Shp1 anti-invasive effects, unlike that of GroPIns4*P* that was still active (about 40% inhibition) (Fig. 4B and Additional file 1: Figure S3).

Next, we investigated whether Shp1 was able to directly bind GroPIns (as it was demonstrated for GroPIns4*P* [16]). In in vitro pull-down assays purified recombinant Shp1 was specifically pulled-down by GroPIns-Bio-bound beads (and not by control Biotin-bound beads; see “Methods” section), indicating that GroPIns binds directly to Shp1 (Fig. 4C). We then determined the region of Shp1 involved in this interaction by evaluating the binding of GroPIns to two previously-characterized Shp1-truncated fragments (Fig. 4D; [16]). We found that only the His-SH2 (N + C) mutant that comprises the N-terminal portion composed of the two SH2 domains retains the ability to bind GroPIns, while the central catalytic domain (His-PTPase) did not (Fig. 4C). These data indicate that GroPIns, like GroPIns4*P*, binds to the N-terminal portion of the protein and does not interact with the phosphatase domain of the enzyme.

Collectively, these results define GroPIns as a direct modulator of the Shp1 inhibition of matrix degradation in A375MM cells.

### GroPIns facilitates Shp1-cortactin association

Having shown that GroPIns is able to modulate the Shp1-dependent inhibition of A375MM melanoma cell ECM degradation, we tested whether this modulation could involve Shp1 activity on cortactin. Since we showed previously that GroPIns4*P* increases the affinity of Shp1 for substrates such as Src [16], we hypothesized that GroPIns could also affect the Shp1-cortactin interaction.

**Fig. 5:**
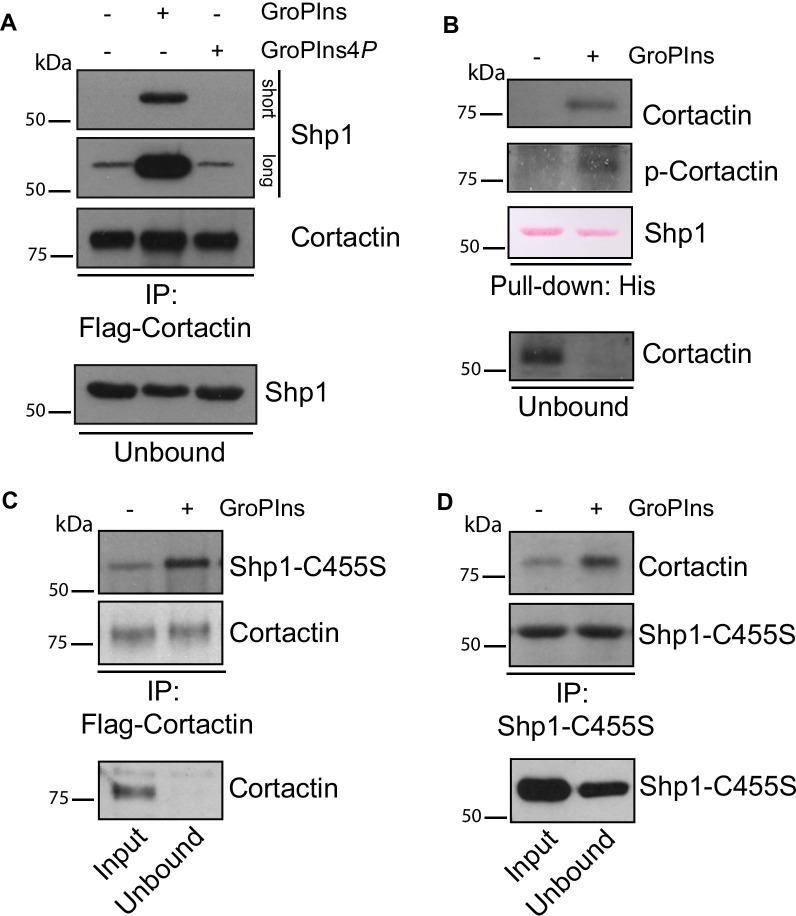
GroPIns favors the association between Shp1 and cortactin. **A** Representative pull-down assay of His-Shp1 with Flag-cortactin-beads from A375MM lysates over-expressing Flag-cortactin. Beads were treated with buffer alone (−) or with GroPIns and GroPIns4*P* (as indicated), and then incubated with purified His-Shp1. The eluted and unbound proteins were analyzed by western blotting using an anti-Shp1 antibody, while immunopurified Flag-cortactin was revealed using an anti-cortactin antibody. Shp1 staining is shown with two different exposure times (short and long) for better interpretation. **B** Representative in vitro pull-down assay of cortactin, previously phosphorylated on tyrosine residues by Src kinase in an in vitro kinase assay, with His-Shp1. The unbound and eluted proteins were analyzed by western blotting using anti-phosphotyrosine and anti-cortactin antibodies. His-Shp1 was revealed by Ponceau staining. **C**, **D** Interaction between Shp1 C455S mutant and cortactin was examined by immunoprecipitation (IP) with an anti-Flag (**C**) or anti-Shp1 (**D**) antibodies in A375MM cells over-expressing Shp1 C455S mutant and Flag-cortactin, untreated (−) or treated (+) with 50 μM GroPIns for 30 min (as indicated). Representative western blotting with anti-Shp1 and anti-cortactin antibodies, of total lysate (input), unbound and immunoprecipitated proteins (as indicated). Molecular weight standards (kDa) are indicated on the left of each panel. The blots shown are representative of at least two different experiments

To investigate this aspect, in vitro pull-down assays were performed using purified recombinant Shp1 and cortactin (see “Methods” section), but no association between these proteins could be detected, either in absence or presence of GroPIns (Additional file 1: Figure S4). Having shown that Shp1 dephosphorylates cortactin in vitro (Fig. 3B), we evaluated whether cortactin phosphorylation was required for its association with Shp1. To this end, we examined the association of immobilized Flag-tagged cortactin immunoprecipitated from A375MM cells (thus, endowed with the post-translational modification occurring in mammalian cells, but not in recombinant-bacterial proteins; see “Methods” section) with Shp1 in the presence of GroPIns, and observed a pronounced binding as compared to that in control (buffer only) or in the presence of GroPIns4*P* taken here as a negative control (and similar to buffer; Fig. 5A).

Based on the above results, we conclude that the interaction between Shp1 and cortactin requires the latter to be phosphorylated. To further substantiate this finding, we examined whether the tyrosine phosphorylation of cortactin was a determinant in its interaction with Shp1. The three tyrosines known to be phosphorylated downstream of Src are residues 421, 466, and 482 of mouse cortactin [45, 46]. Thus, purified recombinant cortactin was subjected to an in vitro kinase assay with recombinant Src prior to its use in pull-down assays with recombinant Shp1, both in absence and presence of GroPIns (see “Methods” section). Under these conditions, GroPIns induced the association of the two proteins, confirming that Shp1-cortactin interaction requires the phosphorylation of cortactin on tyrosine residues (Fig. 5B).

The modulation of the Shp1-cortactin interaction by GroPIns was also observed in intact A375MM cells. Since the interaction of wild-type Shp1 with its substrates is known to be transient [47], we overexpressed the Shp1-C455S-trapping mutant (as it forms stable, covalently-linked complexes with phosphorylated target proteins [48]) and observed an increase in the formation of the Shp1-C455S-cortactin complex when the cells were treated with GroPIns (shown by coimmunoprecipitation; see “Methods” section and Fig. 5C, D).

From all the above results we concluded that only the phosphorylated form of cortactin binds to Shp1 and that this interaction is facilitated by GroPIns.

### The Shp1-cortactin association is at the invadopodia site

**Fig. 6:**
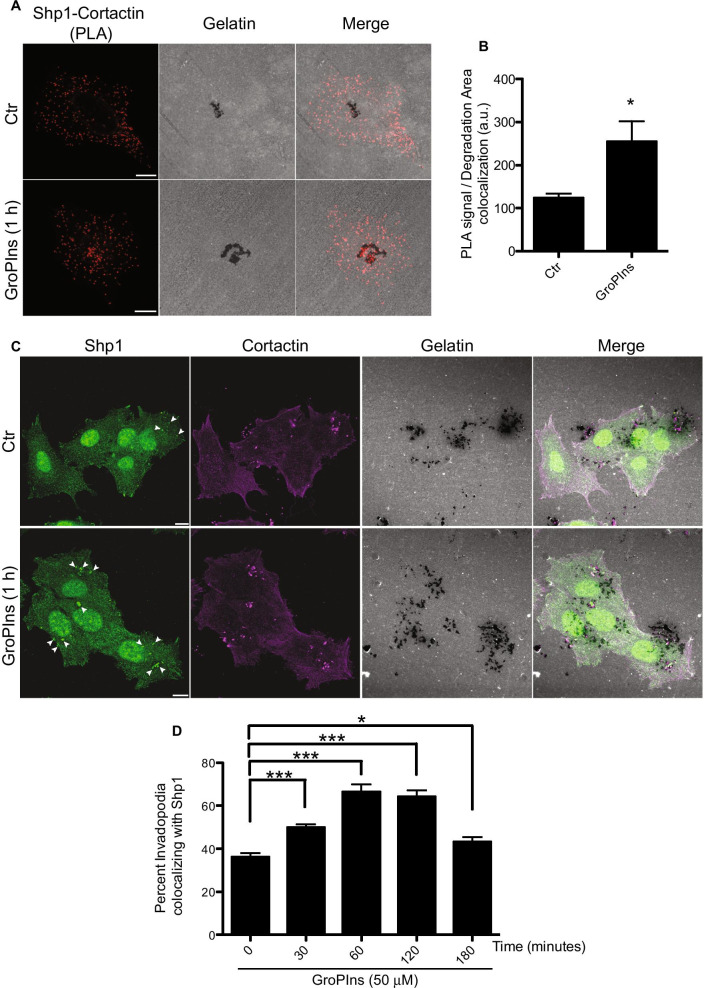
GroPIns facilitates Shp1 localization and association with cortactin at invadopodia. **A** Representative images of PLA assay carried out on A375MM cells cultured on fluorescent gelatin-coated coverslips and then treated with 50 μM GroPIns for 1 h (see “Methods” section). Cells were fixed and stained with antibodies against endogenous Shp1 (rabbit polyclonal antibody) and endogenous cortactin (monoclonal murine antibody), followed by incubation with PLA probes minus and plus, ligation and amplification. PLA signal (red spots) indicates Shp1-Cortactin complexes. **B** Quantification of PLA signal/Degradation area colocalization (expressed as percentages of control) of cells treated as in **A**. Data are expressed as the means (± SE) of two independent experiments performed in duplicate. **C** Representative confocal images of A375MM cells grown on fluorescent gelatin-coated coverslips for 16 h and then untreated (Ctr) or treated with 50 µM GroPIns for 1 h. The cells were fixed and stained for shp1 and cortactin (following cytosol extraction as described in “Methods” section). Shp1, cortactin and gelatin are shown in green, magenta and gray, respectively. The degraded ECM is shown as dark areas and refers to the degradative activity resulting from ~ 17 h of incubation of cells on the gelatin. Invadopodia present following 1 h of GroPIns treatment are shown as actin-positive dots corresponding to the dark areas. Arrowheads indicate invadopodia colocalizing with Shp1. **D** Quantification of invadopodia colocalizing with Shp1 (expressed as percentage of total cells) of cells treated with 50 µM GroPIns for the indicated times. Data are expressed as the means (± SE) of three independent experiments performed in duplicate. ***P < 0.001; *P < 0.05 (Student’s *t*-test) calculated for each treatment *versus* untreated samples. Scale bars, 10 μm

As cortactin is needed for invadopodia development, it was reasonable to hypothesize that the Shp1 catalytic activity may control this process. A way to investigate this aspect was to evaluate whether GroPIns, that modulates the Shp1-cortactin interaction, affects invadopodia formation. Thus, A375MM cells were plated on FITC-labelled gelatin for 16 h and examined with the *in-situ* proximity ligation assay (PLA, see “Methods” section [49]) with Shp1, cortactin or IgG antibodies, the last ones as negative control. The colocalization of PLA signals with area of matrix degradation was quantified to derive the extent of the Shp1-cortactin interaction related to invadopodia formation, in both untreated and GroPIns-treated (1 h) cells (Fig. 6A). It should be noted that the basal matrix-degrading activity occurring during the overnight incubation of cells on the gelatin and prior to the addition of GroPIns can be visualized by the degraded area observed in control cells; the same is then part of the degradation observed also after GroPIns treatment. The results shown refer to the interaction and activity of Shp1 and cortactin during this time (1 h); indeed, Shp1-cortactin associating at the degradation area yielded a twofold increase in PLA signal after GroPIns treatment, as compared to untreated cells, confirming that the interaction of these two proteins occurs at the invadopodia area (Fig. 6B).

At this point we investigated whether the role of GroPIns in facilitating the Shp1-cortactin interaction was specifically exerted at the invadopodia. By immunofluorescence analysis, Shp1 was found to localize at invadopodia in approximately 40% of the A375MM cells examined and this value increased to about 75% following GroPIns treatment (Fig. 6C, D).

In summary, these results indicate that the Shp1-cortactin association occurs preferentially at the invadopodia sites and is facilitated by the presence of GroPIns.

### GroPIns-Shp1-cortactin-dependent inhibition of invadopodia function

Since GroPIns promotes the interaction of Shp1 with cortactin at invadopodia, we investigated whether its inhibitory role on invadopodia function could involve the induction of cortactin dephosphorylation on tyrosine 421. This could be the cause of the disassembly of the protein complex with phosphorylated cortactin as scaffold, required for invadopodia formation.

**Fig. 7:**
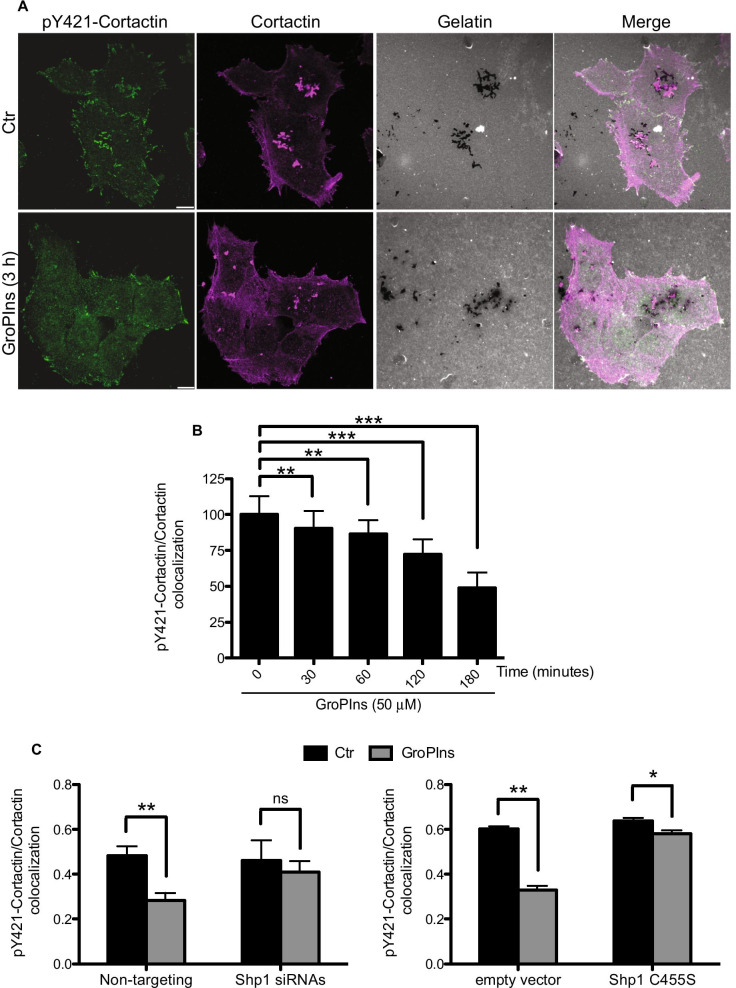
GroPIns promotes Shp1-mediated dephosphorylation of cortactin. **A** Representative images of A375MM cells plated on fluorescent gelatin-coated coverslips and then untreated (Ctr) or treated with 50 µM GroPIns for 3 h. Cells were fixed and stained for pY421-cortactin and cortactin. pY421-cortactin, cortactin and gelatin are shown in green, magenta and gray, respectively. The degraded ECM is shown as dark areas and refers to the degradative activity resulting from ~ 19 h of incubation of cells on the gelatin. Invadopodia present following 3 h of GroPIns treatment are shown as actin-positive dots corresponding to the dark areas. **B** Quantification of cortactin phosphorylation (expressed as percentage of control) of cells treated with 50 µM GroPIns for the indicated times. Cortactin phosphorylation was expressed as the fold change in pY421-cortactin/cortactin ratio localized to invadopodia. Data are expressed as the means (± SE) of three independent experiments. **C** Quantification of cortactin phosphorylation of non-targeting siRNAs and Shp-1 siRNAs-treated A375MM cells (left panel) and of empty vector and Shp1 C455S transfected cells (right panel) left untreated (Ctr) or treated with 50 µM GroPIns for 3 h. Data are expressed as the means (± SE) of two independent experiments performed in duplicate. ***P < 0.001; **P < 0.02; *P < 0.05; ns *P* > 0.05 (Student’s *t*-test) calculated for each treatment *versus* untreated samples. Scale bars, 10 μm

Indeed, in matrix degrading A375MM cells, pY421-cortactin, evaluated by immunofluorescence, was enriched at the leading edge of lamellipodia and at invadopodia (Fig. 7A), and the number of invadopodia containing pY421-cortactin, evaluated by immunofluorescence analysis, was significantly reduced following GroPIns treatment (Fig. 7B). Importantly, the kinetic of pY421-cortactin decrease paralleled the GroPIns-induced recruitment of Shp1 at invadopodia (see Fig. 6D), suggesting that the chain of event leading to invadopodia formation (hence ECM degradation) requires the phosphorylation of pY421-cortactin that contributes to the formation of the invadopodia structure together with other structural proteins (as discussed above; [50]).

The role of the phosphatase activity of Shp1 in the above scheme was examined by transiently knocking it down in A375MM cells; cortactin phosphorylation was then assayed in both untreated and GroPIns-treated cells. Consistent with the ECM degradation data described in previous paragraphs, the levels of pY421-cortactin in Shp1-depleted cells were not significantly affected following GroPIns treatment, supporting the notion that Shp1 is required for cortactin function and that GroPIns-represent a regulatory element in this process (Fig. 7C, left panel). Similar data were obtained in Shp1-C455S-overexpressing cells, i.e., under conditions in which Shp1 could not dephosphorylate cortactin (Fig. 7C, right panel).

A conclusion deriving from the above data is that in A375MM melanoma cells phosphorylated cortactin is necessary but not sufficient to promote invadopodia dynamics, in that Shp1 and GroPIns are necessary complements for this event (see above). It is therefore conceivable that the complex of proteins associated with cortactin to form the invadopodia structure also requires Shp1. Along the same line of evidence, the phosphorylation of cortactin on tyrosine 421 is necessary for the release of cofilin from cortactin, and the recruitment of the N-WASp-activator Nck1 to invadopodia, both events relevant for actin polymerization, invadopodia maturation and ultimately tumor cell invasion [42, 44]. The Shp1-dependent dephosphorylation of cortactin could therefore play a role in the recruitment of these proteins at invadopodia. We thus analyzed if this was the case for Nck1, by examining its colocalization with cortactin in Shp1-knockdown cells. Surprisingly, this colocalization was reduced only in non-targeting siRNAs-transfected cells treated with GroPIns (Additional file 1: Figure S5A); conversely, GroPIns completely failed to have any effect in Shp1-knockdown cells (Additional file 1: Figure S5B, left panel). Similar results again were observed in Shp1-C455S-overexpressing cells, strengthening the role of the Shp1 phosphatase activity in this process (Additional file 1: Figure S5B, right panel).

Taken together, these experiments identify Shp1 as an upstream regulator of cortactin and demonstrate that the GroPIns-induced dephosphorylation of cortactin is a key event to modulate invadopodia function and matrix proteolysis.

### GroPIns inhibits tumor invasion in vivo

**Fig. 8:**
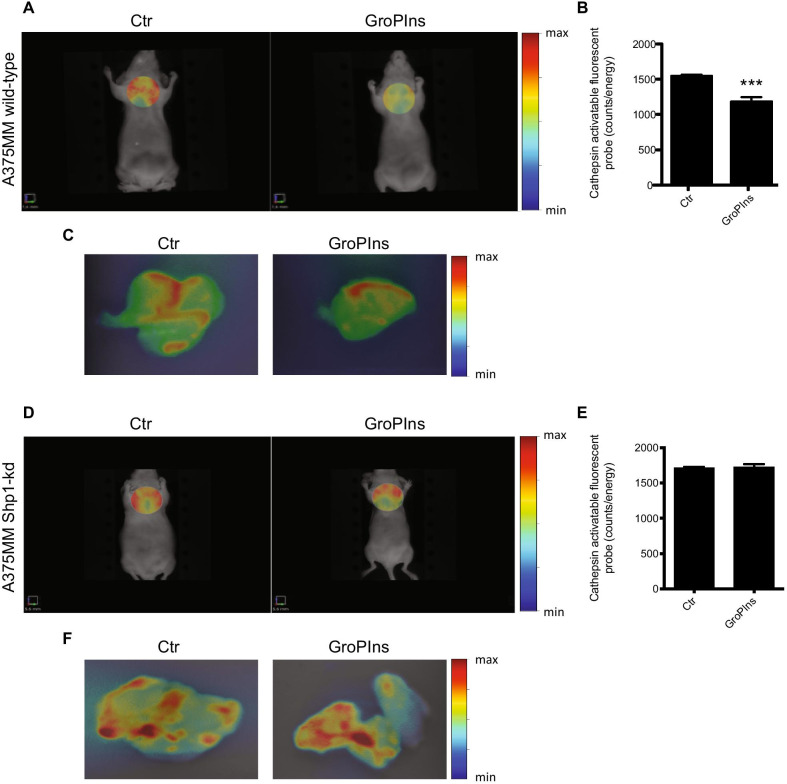
In vivo imaging of lung metastases in a A375MM wild-type and Shp1-kd mouse models after GroPIns treatment. **A** Representative images of fluorescence reflectance imaging (FRI) of BALB/c nude mice bearing A375MM-derived lung metastases treated with PBS (Control; 6 mice) or GroPIns (50 mg/kg; 6 mice) 5 days/week for 2 weeks. After treatment, mice were i.v. injected with 2 nmol of ProSense 750 and after 24 h they were subjected to imaging studies by FMT 4000. **B** Quantification of Cathepsin activatable fluorescent probe (expressed as counts/energy) of animals treated as in **A**. **C** Representative ex vivo images of the lungs excised from control and GroPIns-treated mice after in vivo imaging. **D** Representative images of FRI of BALB/c nude mice bearing A375MM Shp1-kd-derived lung metastases treated with PBS (Control; 4 mice) or GroPIns (50 mg/kg; 4 mice) 5 days/week for 2 weeks. After treatment, mice were i.v. injected with 2 nmol of ProSense 750 and after 24 h they were subjected to imaging studies by FMT 4000. **E** Quantification of Cathepsin activatable fluorescent probe (expressed as counts/energy) of animals treated as in **D**. **F** Representative ex vivo images of the lungs excised from control and GroPIns-treated mice after in vivo imaging. ***P < 0.001 (Student’s *t*-test) calculated for each treatment versus untreated samples

Collectively, the data presented in this study indicate that the naturally occurring compound GroPIns upon binding to Shp1 has the potential to interfere with the ability of melanoma cells to invade the ECM and thus promote metastases. On this basis and considering the low-molecular-weight, water-solubility and non-toxic proprieties of this compound [51, 52], we evaluated its activity upon administration in in vivo studies (see “Methods” section)*.* Thus, we examined the anti-invasive efficacy of GroPIns in a metastastic model of melanoma, established by tail-vein injection of A375MM cells in BALB/c nude mice [50]. After two weeks of treatments (50 mg/kg for 2 weeks; see “Methods” section), the control and GroPIns treated-mice were subjected to fluorescent molecular tomography with ProSense 750, a cathepsin-activatable fluorescent imaging agent, commonly used to detect the metastatic process [50, 53]. As shown in Fig. 8A, an intense NIR fluorescence signal using optical FRI was detected in lung metastases 24 h after ProSense 750 injection in control mice. A significant reduction of ProSense 750 uptake (≈ 25% reduction) was observed in the lungs of mice that received GroPIns treatment compared to those treated with vehicle alone (Fig. 8B). This observation was supported by the ex vivo images of lungs (see “Methods” section; Fig. 8C), where the fluorescence signal was reduced following GroPIns treatment.

Additional evidence in support of the involvement of Shp1 in the GroPIns control of the metastatic dissemination was obtained by using a A375MM Shp1-knockdown cell line that we generated by CRISPr/Cas9-mediated genome editing. These cells, showing a significant decrease in Shp1 expression levels (≈ 80% reduction; Additional file 1: Figure S6 A-B), were first validated in in vitro ECM degradation assays and found to be completely unresponsive to GroPIns treatment (Additional file 1: Figure S6 C-D). Then, cells were injected in the tail-vein of BALB/c nude mice and the anti-invasive efficacy of GroPIns was evaluated following two weeks of treatments (50 mg/kg for 2 weeks; see “Methods” section). As shown in Fig. 8D, E, no significant reduction of ProSense 750 uptake was observed in the lungs of mice that received GroPIns treatment as compared to control animals (treated with vehicle alone), thus confirming the crucial role of Shp1 in GroPIns-effect on cancer invasion.

Altogheter, these in vivo data support the finding that the GroPIns-Shp1-cortactin complex by inhibiting invadopodia formation, may affect the invasiveness of melanoma cells in an in vivo model system.

## Discussion

**Fig. 9:**
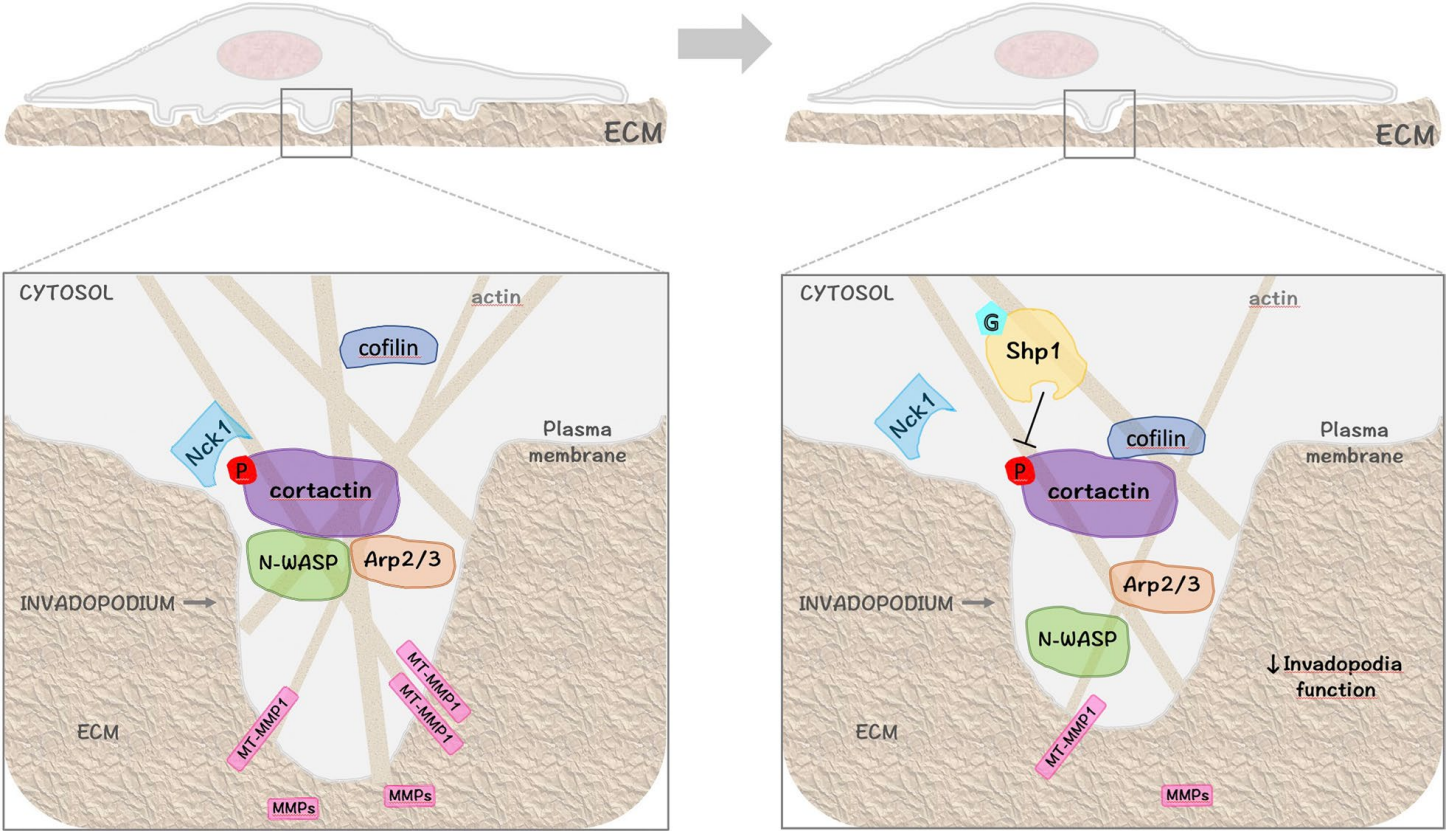
Model of Shp1-dependent regulation of invadopodia. Schematic representation of an A375MM melanoma cell with ventral invadopodia degrading the extracellular matrix (ECM). Cortactin phosphorylation on tyrosine 421 results in disruption of the inhibitory interaction between cortactin and cofilin with the recruitment and activation of multiple components of the actin regulatory machinery (N-WASP, Arp2/3, Nck1) to promote actin polymerization, matrix-metalloproteasis (MMP) recruitment and efficient invadopodial matrix degradation. The GroPIns (G) binds to Shp1 and induces its localization at invadopodia. This leads to cortactin dephosphorylation at tyrosine 421, with a consequent impairment of cortactin scaffolding-activity, invadopodia disassembly and reduced cancer cell invasion. See text for details

Degradation of ECM is a critical step for the invasion of cancer cells that are generally surrounded by dense basement membranes. Increasing evidence indicates that invasive cells digest these membranes by making small perforations that are the results of an efficient invadopodia activity [2]. In this study, we have identified the tyrosine phosphatase Shp1 as a novel signaling component involved in the regulation of ECM degradation through its ability to govern invadopodia formation and activity. This conclusion is based on the following findings: 1. Shp1 localizes at invadopodia in A375MM melanoma cells; 2. Shp1 interacts with cortactin and reduces cortactin phosphorylation at tyrosine 421; 3. induction of Shp1-cortactin complex formation impairs cortactin scaffolding-activity and negatively affects invadopodia behaviour; 4. the natural compound GroPIns by directly binding Shp1, facilitates its interaction with, and dephosphorylation of, cortactin giving rise to the active complex GroPIns-Shp1-cortactin that inhibits invadopodia function (Fig. 9).

Invadopodia are very dynamic membrane protrusions, whose function is tightly regulated by both cytoskeleton and signaling proteins [25]. However, how protein complexes are assembled into a functional unit is still not completely understood. Recent data indicate that the interactions among scaffold proteins determine the formation of signalosomes to coordinate events that regulate actin cytoskeleton dynamics and functions; in this context cortactin is considered a master signalosome for the plethora of cortactin-interacting proteins [54].

Cortactin over-expression is common to several cancer types, and is associated with enhanced motility, invasion and invadopodia activity [55]. Recruitment of cortactin is indeed necessary for invadopodia initiation, where phosphorylation of C-terminal tyrosines 421, 470, and 486, along with serines 405 and 418, occurs downstream of growth factors and integrin signaling [56]. These phosphorylation events are essential for cellular invasion and distal tumor metastases formation through multiple mechanisms ultimately creating binding sites for scaffolding platforms composed by proteins such as Arp2/3, cofilin, N-WASp, Grb2, Nck1 and others [41–43, 57, 58]. Specifically, phosphorylation at tyrosine 421 is essential for Nck1 binding to cortactin and the subsequent Nck1–N-WASp–Arp2/3 complex assembly, which is required for efficient actin polymerization within invadopodia [43].

Here we identify a specific interaction between cortactin and the tyrosine phosphatase Shp1, demonstrating a novel role for Shp1 at invadopodia. We show that Shp1 directly binds to the tyrosine-phosphorylated cortactin and dephosphorylates it specifically at tyrosine 421. This impairs cortactin function and presumably negatively affects invadopodia stability. An increased invadopodia activity is indeed observed in A375MM cells with the overexpression of the catalytically inactive Shp1 mutant or siRNAs-mediated Shp1 knockdown.

Although Shp1 was previously reported to modulate Src kinase activity by dephosphorylation of the Src-inhibitory phosphotyrosine in position 530 [59], this was not the case in A375MM cells. By co-immunoprecipitation experiments, we show that in these cells Shp1 does not interact with Src but rather with its substrate cortactin. This observation is in agreement with the notion that substrates that are efficiently phosphorylated by Src kinase are in turn efficient substrates for Shp1 [60]. We also show that Shp1 acts as a negative regulator of invadopodia in A375MM cells; apparently this is not in agreement with the positive role that has been reported for Shp1 on Src kinase activity in other cellular systems [6]. But Src is also known to play an essential role in triggering invadopodia formation by phosphorylating and activating key structural components of invadopodia, including Tks5 and cortactin [46]. Thus, the role of kinases such as Src or phosphatases needs to be thought of considering the context of the cell system or cancer cells of interest.

An additional novel aspect of our study is the regulation of Shp1 activity by the phospholipid-derived mediator GroPIns. The glycerophosphoinositols are biologically active metabolites that arise from cPLA_2_α activity on the membrane phosphoinositides [61]. Like their parental phosphoinositides, the glycerophosphoinositols exist in several phosphorylated forms within cells, although the non-phosphorylated form (GroPIns) and the form phosphorylated in position 4 on the inositol ring (GroPIns4*P*) are the most abundant and well-studied [51]. Here we show that when exogenously added, GroPIns binds to Shp1 and promotes Shp1-cortactin interaction within invadopodia. This interaction results in the specific dephosphorylation of tyrosine 421 of cortactin, and the subsequent reduction of cortactin-mediated recruitment of the adaptor protein Nck1. This event affects invadopodia formation, and as a consequence, leads to reduction of the ECM degradation in melanoma cells.

A proteomic study identified Shp1 as the direct-molecular target of both GroPIns and GroPIns4*P* [16]. Shp1 however is not part of the signaling induced by GroPIns4*P* to inhibit ECM degradation in A375MM cells; indeed, the depletion of Shp1 in this system completely abrogates the GroPIns-mediated inhibition of tumor invasiveness while does not affect this GroPIns4*P* function. This observation suggests that the molecular cascades initiated by GroPIns and GroPIns4*P* downstream Shp1 diverge, possibly by recruiting different interactors that lead to different biological effects.

The GroPIns-induced regulation of invadopodia function discussed so far refers to melanoma cells. We could envision that a similar control could take place under physiological conditions. We have previously reported that the cytosolic form of PLA_2_ (cPLA_2_α) catalyzes the formation of the glycerophosphoinositols in different cell types upon hormonal stimulation [61] and, in particular, that the EGF-receptor-dependent cell motility requires the cPLA_2_α-induced GroPIns4*P* increase and binding to Shp1 [16]. In a parallel manner, an endogenous increase of GroPIns at the invadopodia site could physiologically regulate the assembly/disassembly of invadopodia. This is an appealing hypothesis that requires the cPLA_2_α localization compatible with the in situ formation of GroPIns. Indeed, in preliminary immunofluorescence experiments cPLA_2_α was localized at the invadopodia site in its phosphorylated, active form (Additional file 1: Figure S7) indicating that it is in a close vicinity of Shp1 and cortactin and could, therefore, endogenously form GroPIns, favoring the interaction between these two proteins as discussed above. How the control of Shp1-cortactin interaction is regulated under physiological conditions however requires further work and is out of the scopes of the present study.

Invadopodia formation, ECM degradation and the ensuing tumor spreading are finely regulated by specific signaling cascades that can share some elements, as is the case of Shp1 that, beside regulating invadopodia function, is a suppressor of TGF-β1-triggered EMT and of metastases through the dephosphorylation of STAT3 [26, 27]. Shp1 has also been shown as a tumor suppressor that negatively regulates cell signaling and cell growth in a variety of cancers [10, 62]. Accordingly, a low expression of Shp1 has been observed in aggressive tumors and has been associated with increased invasive capacity [63, 64], although its role in cell invasion is still poorly understood. Several drugs already used in chemotherapy to suppress tumor growth, including sorafenib [65], dovitinib [66] and the Mcl-1 inhibitor SC-2001 [67, 68], exert their antitumor effects by enhancing the phosphatase activity of Shp1 on the transcriptional factor STAT3 [28]. In this study we show that the Shp1 activity exerted on cortactin is enhanced by the natural compound GroPIns. This molecule was effective in vitro and also in vivo where it partially inhibited the development of cancer metastases in a model of melanoma.

The control of the metastatic cancer dissemination that accelerates patient decline represents a main hindrance in the clinical treatment of cancer. Induction of Shp1 activity by GroPIns treatment might provide support for the utility of GroPIns in cancer treatment. More effective treatment options are indeed required for advanced-stage cancer patients. A possible strategy is the simultaneously inhibition of multiple targets or pathways regulating processes that promote tumor development. A case in point could be the GroPIns that might be combined with other drugs to enhance its clinical efficacy. Moreover, successful therapies require identification of agents that, when combined, lead to synergistic tumor inhibition without significant systemic toxicity. In this context, the GroPIns being a natural compound that is present in virtually all cell types, could be thought of as an adjuvant/cooperator in anti-cancer strategies. Nevertheless, these are proposals that require further evaluation in in vivo models of cancer disease and dissemination.

## Conclusions

This study emphasizes two novel aspects relevant to ECM degradation: first the definition of the role of Shp1 in invadopodia dynamics; second, the elucidation of the molecular mechanisms involved in this event, *i.e.,* the dephosphorylation of cortactin, and the possibility to enhance this modification by the use of the bioactive compound GroPIns. Since the cellular target/receptor of GroPIns is Shp1, these molecules are both of interest for further pharmacological exploitations. Indeed, Shp1 can now be considered as a target for antimetastatic treatment, while GroPIns could be a lead for further drug development.

## Supplementary Information

**Additional file 1: Figure S1**. Shp1 does not dephosphorylate Src on tyrosine 416. **Figure S2**. Shp1 mediates GroPIns-induced inhibition of ECM degradation. **Figure S3**. The enzymatic activity of Shp1 is required for GroPIns-induced inhibition of ECM degradation. **Figure S4**. Shp1 does not bind to unphosphorylated cortactin. **Figure S5**. GroPIns reduces Nck1 localization to invadopodia through Shp1 activity. **Figure S6**. Generation of the stable Shp1-knockdown cell line. **Figure S7**. Active cPLA2 localizes at invadopodia in A375MM cells.

## Data Availability

All data generated or analysed during this study are included in this published article and its supplementary information files.

## Abbreviations

ECM: Extracellular matrix
MMPs: Matrix metalloproteases
EMT: Epithelial–mesenchymal transition
PTKs: Protein tyrosine kinases
PTPs: Protein tyrosine phosphatases
SH2: Src-homology 2
Shp1: SH2 domain-containing phosphatase 1
GroPIns4*P*: Glycerophosphoinositol 4-phosphate
GroPIns: Glycerophosphoinositol
FRI: Fluorescent reflectance imaging
PLA: Proximity ligation assay
cPLA_2_: Cytosolic-phospholipase A_2_
