# Supplementary material for: The phosphatase Shp1 interacts with and dephosphorylates cortactin to inhibit invadopodia function
Source: Cell Commun Signal. 2021 Jun 4;19:64. doi: 10.1186/s12964-021-00747-6 (PMC8176763; doi:10.1186/s12964-021-00747-6)
Supplement: Supplementary file 2 — Additional file 1: Figure S1. Shp1 does not dephosphorylate Src on tyrosine 416. Figure S2. Shp1 mediates GroPIns-induced inhibition of ECM degradation. Figure S3. The enzymatic activity of Shp1 is required for GroPIns-induced inhibition of ECM degradation. Figure S4. Shp1 does not bind to unphosphorylated cortactin. Figure S5. GroPIns reduces Nck1 localization to invadopodia through Shp1 activity. Figure S6. Generation of the stable Shp1-knockdown cell line. Figure S7. Active cPLA2 localizes at invadopodia in A375MM cells. [file 12964_2021_747_MOESM2_ESM.docx]

**Varone *et al*	SUPPLEMENTARY INFORMATION**

**Figure S1: Shp1 does not dephosphorylate Src on tyrosine 416.**

Representative immunoprecipitated Src fraction (IP: Src) from A375MM cell lysates washed and incubated with purified recombinant Shp1 (2 µg) for the indicated time points at 37 °C in the absence (−) or presence (+) of 5mM Na_3_VO_4_ (as indicated). The tyrosine-phosphatase inhibitor Na_3_VO_4_ was used as negative control. The top panel shows western blot analysis with an anti-pY416-Src antibody that reveals the specific phosphorylation of tyrosine at position 416 in Src; this is not affected by the addition of the phosphatase Shp1. The blot was then re-probed with an anti-Src antibody for the identification of immunoprecipitated protein (*middle panel*). A second, lower band was also revealed by the anti-Src antibody, due to a cross-reaction with Shp1, added to the sample as purified protein. The cross-reaction with His-Shp1 was confirmed by Ponceau staining (*lower panel*). Molecular weight standards (kDa) are indicated on the left of each panel. IP, immunoprecipitation.

**Figure S2: Shp1 mediates GroPIns-induced inhibition of ECM degradation.**

Representative confocal images of non-targeting siRNAs (A) and Shp1 siRNAs-treated (B) A375MM cells plated on fluorescent gelatin-coated coverslips (gray) and incubated in the absence and presence of 50 μM GroPIns and GroPIns4*P* for 16 h. Cells were then fixed and stained with phalloidin (magenta). Degraded ECM is shown as dark areas on gray gelatin. The invadopodia are shown as actin-positive dots corresponding to the dark areas. Scale bars, 10 μm.

**Figure S3: The enzymatic activity of Shp1 is required for GroPIns-induced inhibition of ECM degradation.**

Representative confocal images of untransfected A375MM cells (A) or transfected with Shp1 WT (B) or the Shp1 C455S mutant (C) and plated on fluorescent gelatin-coated coverslips (gray) and incubated in the absence and presence of 50 μM GroPIns and GroPIns4*P* for 16 h. Cells were fixed and stained with an anti-Shp1 antibody (green) and phalloidin (magenta). Degraded ECM is shown as dark areas on gray gelatin. The invadopodia are shown as actin-positive dots corresponding to the dark areas. Scale bars, 10 μm.

**Figure S4: Shp1 does not bind to unphosphorylated cortactin.**

Representative *in-vitro* pull-down assay of GST-cortactin with His-Shp1. GST-cortactin conjugated beads were treated with buffer alone (−) or with GroPIns (+) and then incubated with purified His-Shp1. The eluted and unbound proteins were analyzed by western blotting using anti-cortactin and anti-Shp1 antibodies (as indicated). No binding of His-Shp1 to GST-cortactin was observed. Molecular weight standards (kDa) are indicated on the left of each panel. The blots shown are representative of at least two different experiments.

**Figure S5: GroPIns reduces Nck1 localization to invadopodia through Shp1 activity.**

(A) Representative images of A375MM cells plated on fluorescent gelatin-coated coverslips and then untreated (Ctr) or treated with 50 µM GroPIns for 3 h. Cells were fixed and stained for Nck1 (green), cortactin (magenta) and actin (gray). Arrowheads indicate invadopodia colocalizing with Nck1. (B-C) Quantification of Nck1/cortactin colocalization, in non-targeting siRNAs and Shp-1 siRNAs-treated A375MM cells (B) and of empty vector and Shp1 C455S transfected cells (C) left untreated (Ctr) or treated with 50 µM GroPIns for 3 h. Data are expressed as the means (±SE) of two independent experiments performed in duplicate. ****P*< 0.001; ***P*< 0.02; **P* < 0.05; ns *P* > 0.05 (Student’s *t*-test) calculated for each treatment *versus* untreated samples. Scale bars, 10 μm.

**Figure S6: Generation of the stable Shp1-knockdown cell line.**

(A) Western blot analysis of Shp1 protein in A375MM Shp1-kd cell line generated by CRISPr/Cas9 system. Tubulin was used as a loading control. Molecular weight standards (kDa) are indicated on the left of each panel. (B) qRT-PCR showing the expression of *ptpn6* gene in A375MM Shp1-kd cell line generated by CRISPr/Cas9 system. The samples were normalized to the housekeeping gene GAPDH. (C) Representative confocal images of wild-type and Shp1-kd A375MM cells plated on fluorescent gelatin-coated coverslips (gray) and incubated in the absence and presence of 50 μM GroPIns for 16 h. Cells were then fixed and stained with phalloidin (magenta). Degraded ECM is shown as dark areas on gray gelatin. (D) Quantification of the total area of degradation (expressed as percentages of control; at least 30 cells were analyzed for each condition) of cells treated as in C. Data are expressed as the means (±SD) of two independent experiments performed in duplicate. ***P* < 0.02; **P* < 0.05; ns *P* > 0.05 (Student’s t-test) calculated for each treatment *versus* untreated samples. Scale bars, 10 μm.

**Figure S7: Active cPLA_2_ localizes at invadopodia in A375MM cells.**

Representative confocal images of A375MM cells showing colocalization of active pS505-cPLA_2_ with invadopodia markers. Cells were plated on fluorescent gelatin-coated coverslips in complete media, fixed and immunostained for pS505-cPLA_2_ and actin. pS505-cPLA_2_, actin and gelatin are shown in green, red and grey, respectively. Degraded ECM is shown as dark areas. The invadopodia are shown as actin-positive dots corresponding to the dark areas. Arrowheads indicate invadopodia colocalizing with pS505-cPLA_2_. Confocal images are representative of at least three different experiments. Scale bars, 10 μm.
